# Dr. Lee’s Reclamation for Dr. Paine

**Published:** 1858-10

**Authors:** 


### *Dr. Lee's Reclamation for Dr. Paine.*

Dr. REESE: I observe in the July number of the "*Nashville Journal of Medicine and Surgery*" an article by the Editor, purporting to be a reply to my defence of Dr. Paine's claim of being the first to point out the nervous mechanism upon which the excito-secretory function depends, and the first and only one who has applied it to pathology and therapeutics. That defence to which the Nashville Journal objects was obligingly published by yourself in the June No. of the American Medical Gazette. But, besides that brief article, the "*New York Journal of Medicine*" for July has a much more extended one upon the same subject, by the same writer. These articles in defence of Professor Paine's claims have been written by myself as a matter of simple justice due him from the medical profession, and because of my long familiarity with his writings. They have also made their appearance in different journals, partly on account of my disinclination to occupy an unreasonable space in one of them, and in part because of the impossibility of condensing the necessary extracts and other proof into one or even two articles that would come within the limits of a medical periodical.

The foregoing, articles, therefore, must be taken in connection by any one who would embark upon the utopian enterprise of disproving the claim to a principle which lies at the foundation of all Dr. Paine's writings. But what is not a little remarkable, the editor of the Nashville Journal touches no part of the proof embraced in the article which he professes to attack, but merely reiterates the piratical premises which it was our object to refute, and calls upon Dr. Paine to say why he had not earlier driven the invaders from his own dominions. This is the entire sum of our critic's effort, and certainly also of his ability, upon the question before us; though I might be unjust to him and myself did I not annex his concluding paragraph, which is generally intended as the most forcible and conclusive part of an argumentative discussion; and, for our mutual benefit, therefore, I shall quote the peroration.

"If we were a bird," says our critic, "we had rather from our own *matrix!* produce an egg; than to sit on all the eggs that had been laid by all the birds since the time they went forth in pairs from Noah's Ark!"

We have only one comment to make upon this elegant metaphor, desiring only to say of it, that it has the misfortune of overlooking the

3

fact that Dr. Paine's writings were before the public many years before Dr. Campbell's egg was laid; from which it follows, by our critic's own showing, that the egg of the latter was stolen from the prolific nest of the former. "It is a bird of ill omen," &c.

We do not, however, regret, except on account of our critic's fair reputation, that he has afforded us an opportunity of extending our proof in behalf of Dr. Paine's priority in all this absurdly controverted matter relative to the organic influences of the nervous system. We say *absurdly*, for we reiterate the fact, as expressed by Dr. Paine in his "Rights of Authors," that "all that has been granted by Dr. Hall to Dr. Campbell, and therefore all that Dr. Allen appropriates to himself, abounds in the "*Institutes*," and, in fact, constitutes the life and soul of the work as it does also of the "*Commentaries*" and the essay "on the *Modus Operandi of Remedies*."

The whole philosophy of the "*Institutes*" published in 1847, and of the essay on the "*Modus Operandi of Remedies*," (1842,) as it respects pathology and therapeutics, the only objects of any practical value, turns essentially upon the influence of the nervous system upon the secretions and excretions, the former of which embraces the entire processes of nutrition, as well as the glandular and other fluid products.

But Dr. Paine regards the *excito-secretory* function of the nervous system as a very minor part of that system, the most important of which is its variously alterative effects upon the organic functions; or, in his own language, "in all the cases the nervous power is rendered *stimulant*, or *depressant*; or *alterative* to the organic properties and functions, and variously energetic, according to the operating cause, and the intensity and suddenness with which it may operate."—P. 107.

Dr. Paine begins his very extended philosophy upon the subject before us by indicating in a general manner the uses and relationship of the two systems of nerves, and we regret we have room only for the following extract:

"The cerebro-spinal nerves and the sympathetic interchange contributions in all parts, by which important influences of the former are established in the organs of organic life. Nevertheless, the cerebro-spinal system is especially designed for the use of animal life; but an important final cause is answered in making it subservient to the common interests of the whole being. The sympathetic system is added especially to the organic life of animals on account of the complexity of the organs, and to unite them in harmonious action through *circles*

of sympathy, and render them, each in its place, conducive to a common end. The cerebro-spinal system contributes to this result; and each system unitedly, or independently, *exerts special influence on the specific actions of organs*, though these actions are carried on essentially through properties inherent in several tissues." "An important consequence of the foregoing union of organic with animal life is a general coincidence in the pathological as well as the physiological condition of the whole The diseases of each react mutually on each system of organs; each requires common methods of treatment, and remedial as well as morbific agents operate upon the universal body through any given organ."—P. 55, and so on to the end of this chapter of 24 pages on "Structure."

It will be our remaining object to present quotations from our author's "Institutes," which, with one or two exceptions, do not appear in our former articles, and such as have a direct bearing upon the strangely controverted subject before us. On coming to our author's analysis of the "Properties of Life," we meet with an extensive display of the influences of the nervous system in its functions of *reflex action*, and where is presented all that can be supposed to be, relative to the excito-secretory system, and that system applied pathologically and therapeutically. Our author's extended disquisition upon this subject of reflex action in organic life is evidently intended as a foundation for all that follows on his copious topics of pathology, therapeutics, blood-letting, and operation of morbific and remedial agents. We shall stop here to repeat one extract, which, in itself, covers the whole ground in dispute, and stretches as far as imagination may please to travel in the regions of pathology and therapeutics. It occurs at p. 108, after having set forth the anatomical medium of the sensitive and excito-motory fibres of the ganglionic and pneumogastic nerves, through which the influences are transmitted to and from the nervous centres.

"The operation of the nervous power," he says, "is excited through the medium of sympathetic sensibility. This complex process results in the true function of sympathy. Impressions are made by physical and moral causes, by disease, &c., upon the foregoing variety of sensibility, and according, also, to its different modifications in different parts, and the nature of the operating causes. The impressions are then communicated to the cerebro-spinal axis, or to other central parts of the nervous system, and there bring into operation and variously modify the nervous power. The power thus developed, thus influenced, or so modified in kind that it partakes of the nature of the transmitted impressions, which are more or less coincident with the virtues of the remote causes, is then exerted through the motor system of nerves, upon the organic properties of distant parts, or of the nervous system itself, by which those properties and their resulting *functions* and *products* are variously affected, according to the foregoing circumstances. From this fact, it also results, that the modified conditions which are brought about by the nervous power when the preternatural operation of this power depends upon external causes, whether morbific or remedial, are more or less analagous to those changes in the organic conditions which are wrought in parts by the direct operations of the same causes."

Among the numerous exemplifications of the foregoing doctrine, and in immediate connection with it, occurs the following:

"Thus, an impression from cold, or a blast of air, or a drop of cold water upon the skin in syncope, will rouse the respiratory organs. Another impression from the same, and under other circumstances, will excite catarrh, pneumonia, or articular rheumatism; one degree of impression upon the stomach by tartarized antimony, will determine the nervous power upon the respiratory muscles, (as will cantharides upon the bladder, and mercury upon the *salivary glands*,) and vomiting is the consequence; while it simultaneously *reflects* the same power upon the skin and other organs, and of which *perspiration*. &c., is a consequence. In smaller doses, the respiratory movements are not affected, but only *the condition of the skin, &c.*, and in lesser degrees. But these examples embrace only certain parts of the influences in each case; while in others they are far more complex, one sympathetic result becoming the cause of others, till, through a single impression upon the skin, various *circles* of morbific or remedial sympathies may be instituted."—P. 108.

The work abounds with similar examples, in many of which, as in the foregoing, the "excito-secretory" function ol the nervous system is brought distinctly before the reader. At page 339 occurs an instance which we shall quote, as it is one of the obvious sources from which the term "excito-secretory" has been derived.

"When the food, as in Section 512, induces vascular action and warmth in the skin before digestion commences, that organ, in consequence, reflects salutary influences upon the digestive organs, and thus promotes digestion. When tartarized antimony, in small doses, establishes its sudorific impression, the skin becomes the source of many sympathetic influences upon other organs; thus showing, also, that it is not the perspiration, but the vital change in the organ itself, which leads to results that cannot be initiated by any other mode of *exciting this excretory function*; and so more or less of other parts upon which the antimony may exert its primary sympathetic effect. Thus it happens, that whether remedial agents are applied to the stomach or skin, sympathetic influences are propagated to each, as well as from each to other organs, while each in its turn reflects the the impression back to the brain and spinal cord, from whence they are again returned with increased intensity."

Now, in the name of common sense, we should like to know what difference there is in the words and their import, "*exciting this excretory function*," as employed in the foregoing quotation, and, "*excito-secretory function*," as employed by Dr. Campbell?"

We might now draw abundantly to the same effect from what our author says in regard to the influenee of the nervous system in excit-citing the secretion of urine as well as of sweat. But we have room for only a single quotation, and we shall present one which shows the operation of the passions, and where the excito motory fibres of the ganglionic nerve supplying secretory organs are alone concerned. Thus, at page 230, we read as follows:

"For the fulfilment of their final cause, the kidneys possess an exquisite susceptibility to the influence of the nervous power. Hence arises the rapid and profuse excretion of urine when fear and certain other emotions of the mind are in operation. The same affirmation, too, may be made of the skin, though perhaps less extensively. This, too, is the reason why fear so readily induces copious sweats. In either case, the phenomena are owing to the *direct* development and determination of the nervous power upon the organs respectively. These phenomena, too, prove the great susceptibllity of the skin and kidneys to the influence of the nervous power, and are a key to the whole philosophy of the interchanges of action between the skin and kidneys."

As a reference was made in the foregoing extract to Section 512, we shall now quote that Section, and in which our author applies the doctrine of reflex action physiologically to a variety of fluid products, and in which, if he does not employ the term "excito-secretory function," he supplies an instance from whence any mere lover of words might readily reduce it. The nervous mechanism had been already distinctly set forth,

"The various nervous communications of the intestinal canal with the brain and all other organs," says Dr. Paine, "are demonstrative of the ascendant influence which the stomach, particularly, possesses, when acted upon by remedial agents. We see all this exemplified, analogically at least, in the endless remote derangements which follow the common irritations and morbid states of the organs, as also of the intestines; we see, indeed, the whole in natural progress. When, for example, hunger operates, an actual sensation is then felt by the brain, and the mind, of course, participates. Numerous and complex influences may be thus brought into operation, of which the stomach is the primary source. The will, being excited, brings into action all those muscles which are necessary to obtain a supply of food, and other muscles to effect its mastication, and convey it to the stomach. Various sympathetic organic influences are in the meantime taking place, many of which spring from the mind itself. Thus, the brain feeling the sensation of hunger, *the salivary glands begin to pour out their fluid* at the sight or smell of food, or even at its expectation. The food establishes an influence upon the nervous centres, by which an exciting *nervous power* is constantly propagated to other parts. The *bile, saliva, &c., are thus increased*, though other more sympathetic influences contribute to these results. The stomach being supplied with its wants, all these influences cease and a new order arises. Cut off the par vagum, and none of them will obtain, unless feebly, through the ganglionic and spinal nerves. When the food has undergone digestion, and all exciting impression is removed from the stomach, all the *reflected influences* of the brain and spinal cord cease in consequence."—P. 335.

The foregoing paragraph is plain, comprehensive, and to the exact purpose before us, and whoever denies it must be wanting either in common understanding or fair dealing. The paragraph also shows our author's manner of applying physiological processes to pathology and therapeutics. He wields with great effect the laws of *reflex* action, as emanating from those portions of the ganglionic nerves and pneumogastric which supply the alimentary canal, and interprets through this medium the influences which are exerted by internal remedies upon remote parts. In entering upon the subject of cathartics, he says:

"It is owing to these vast and important anatomical and physiological connections, that, when disease springs up in the intestinal mucous membrane, it sheds its morbific influence abroad over the whole system; now developing, sympathetically, cerebral inflammation or congestion; now of the liver; again, inflammation of the skin; at another time of the bladder; in this subject, rheumatism; in that, scrofula; in another, croup; in others, inflammation of the fauces; here, of the eyes; there, of the nose; here, an attack of the gout; there, abortion; and so on through every part of the organization. Considering, therefore, I say, the foregoing anatomical and physiological relations, and how diseases of the alimentary mucous tissue may give rise to disease in every other part, we may readily comprehend how it is that cathartics exert powerful effects upon distant organs when rendered unusually susceptible by disease; and so of all other remedial agents, internally applied, according to the nature of their virtue, doses, &c."—P. 565.

Let us now look upon Dr. Paine's long chapter, upon "Remedial Action," and here we shall find numerous exemplifications of the "excito-secretory function," and of the nervous channel through which it is conducted. Here, too, as often before, when entering upon any special topic, Dr. P. begins the disquisition by setting forth his never-failing premises as to the nervous system, and in the following manner:

"We have seen," he says, "that the vital property, sensibility, possesses a modification which I have denominated sympathetic sensibility; that the nervous power is a vital agent, and like other agents developes motion and induces changes by acting upon the organic property, irritability, and is exclusively the exciting cause of motion in animal life; that this power or property of the vital principle in animals may be called in a direct manner into increased or preternatural operation by direct impressions, physical or moral, upon the nervous centres or upon the trunks of nerves; but this power is the efficient agent of remote sympathy, is brought into operation by impressions made upon sympathetic sensibility, which are transmitted by this property of animal life through sensitive nerves to the nervous centres, and their develope the nervous power, which is reflected through motor nerves, upon the irritability of such parts as may be determined by the various influences hitherto expounded, and thus becomes the exciting cause of motion, of morbific and therapeutical changes, &c., in those parts upon which its impressions are made,"—P. 661. There follows immediately a summary application of the foregoing statement to a great variety of physiological, pathological, and therapeutical problems, many of which embrace the "excito-secretary functions," and all of them shown to depend upon reflex or direct action of the nervous system—

the latter being limited to the excito-motory nerves, as when the passions operate, or when impressions are made directly upon the nervous centres; "anatomy and experiment," says our author, "confirm what each phenomenon, and all united proclaim the work of the mystic power operating on those organic properties which are the moving springs of every action, the proximate cause of every effect; nor can another intelligible solution be rendered for a single phenomenon now expressed, or thousands of similar import, while every other must be in conflict with the pronunciations of nature and the demonstrations of art; nor will an attempt be made, (an attempt that shall commend itself to the understanding,) now or hereafter, to controvert the philosophy which is here presented."—P. 663.

This essay on "Remedial action" then takes up a variety of problems in pathology or therapeutics, in ample detail, all of which are interpreted through reflex action, and mainly by the sympathetic and pneumogastric nerves in their connection with the brain and spinal cord. We can only give our readers an apprehension of the manner in which these problems are treated, by a single example, and we select one in which our Nashville critic will find something more about the "excito-secretory function" of the nervous system, and probably to his "heart's content."

"When an emetic operates," says our author, "the modus operandi is essentially similar to what happens in respiration. The mucous tissue of the stomach being the point of departure, a different influence is propagated to the nervous centres, corresponding with the nature of the exciting cause, with the special vital constitution of that portion of the mucous tissue, with the compound nature of the stomach, with the special relations of this organ to the central parts of the nervous system, and to the respiratory muscles, &c.; while the nervous power is also modified in its nature according to the peculiar virtues of the emetic, the most sensible result, as in respiration, depends upon the reflection of the nervous power upon the respiratory muscles, while another current descends through the motor fibres of the pneumogastric and sympathetic nerves to the muscular tissue of the stomach. If the emetic operates also as a cathartic, then a new chain of actions is established, in the same way, upon the abdominal muscles, while a current of the nervous power is propagated upon the muscular coat of the intestines; but, in the foregoing case, something more happens than in the natural processes. Here the exciting cause possesses peculiar virtues, is of a morbific nature, and it not only makes peculiar impressions upon the alimentary mucous tissue, according to the exact nature of its virtues, but it modifies the nervous power in a corresponding manner." Then follows an account of diverse effects, curative or morbific, that may be exerted upon various parts through the medium of nerves with which they are supplied, the same philosophy being thus carried out, that interprets the more sensible result of emetics. Our author then continues his exposition of the alterative influence of reflex action, as developed by emetics. in the following graphic language, and we ask our Nashville critic whether it had before engaged his attention :

" We thus see," continues Dr. Paine, "that when vomiting springs from the operation of tartarized antimony, and often from ipecacuauha, it is only one of the consequences, and a minor one, of the peculiar irritation of the gastro-mucus membrane. Other and far more powerful influences are determined simultaneously upon the organic properties and actions of distant and diseased parts (perhaps as distant as the most remote extremity) by the same nervous power that shook the respiratory organs during the act of vomiting ; and often indeed, does it happen that those influences are propagated with the most profound effect when the act of vomiting fails of being consummated, and nausea alone shall send with prostrating effect the modified nervous power over the whole system ; when we shall see it simultaneously *bathing the whole surface with perspiration ; pouring the saliva from the mouth ;* breaking down a tumultuous excitement of the heart and arteries ; *starting on the instant a torrent of bile. and an equal effusion from the intestinal mucous membrane*, and at the next moment calling up a magnificent play of sympathies for the evacuation of *the fluids* after the manner of an active purgative ; *these very effusions, also, instituting other circles of sympathy,* which join in the great work of curative movements. Should vomiting now follow, then shall you speedily see the vital energies returning ; *the cold, pale skin giving place to a florid hue and a warm perspiration ;* the sunken features starting into the fullness of health ; the gastric suffering gone as a luxury obtained ; the general whirl of anxiety and distress converted into calm tranquillity ; the headache dissipated ; the twang of the croup or the grunt of pneumonia no longer sounding an alarm ; and all this stupendous succession of events, from the beginning of nausea to the restoration of the vital energies and the near resolution of disease, composing a most astonishing consecutive series of sympathies, may require less time than I have hastily employed in this general allusion to the subject. And now can it be entertained that this has been the result of absorption, or that the laws of chemistry or physics have had any connection with the phenomea? The foregoing may be taken as an example of the principle which concerns the *modus operandi* of all curative or morbific agents, whether physical or moral, and of all the developments of disease that arise as sympathetic consequences of each other."—Pages 967–969.

The foregoing paragraph occurs, also, in Dr. Paine's essay on the "*Modus Operandi of Remedies*," published in 1842, which was in the hands of our Nashville critic, as we learn from his journal, at the time he denied Dr. Paine's priority in the matter before us. We shall make no comments on this paragraph. It is more than sufficient for the intelligent reader that we have placed certain parts of it in italics—those parts which refer to the glandular and other fluid products, whence is at once derived, as in preceding quotations, the term "*excito-secretory*," and in which it will be seen that Dr. Paine *does not limit the excito-secretory functions of the nerves to glandular organs*, but extends it to all other parts. But, as we have said, and have shown in this and our other articles, the same proof abounds everywhere in our author's writings; and as to Dr. Allen's double "nervous arc," applied pathologically and therapeutically, and his *exciting and depressing* influence of reflex nervous action, the "Institutes of Medicine" can scarcely be opened at any page from the beginning of the article on "Structure," at p. 50, to the end of "Blood-letting," at p. 777, without finding these doctrines staring him in the face, as the grand elements in all our author's medical philosophy. Should it be said by our Nashville critic that Dr. Paine has not pointed out the particular nerves which supply the glandular organs, we answer that he has been abundantly explicit in specifications of this nature, though he might with great propriety have dispensed with such details; nevertheless, it adds to the value of the "Institutes." As a comprehensive example of our author's manner in this respect, we may quote the following which occurs at page 326:

"Different orders of nerves are concerned in the transmission of impressions, more or less, according to the nature of the exciting causes. Thus, the nerves of volition are not those by which organic processes are influenced. Even in the voluntary muscles the irritability which is relative to their *organic* functions, as also sensibility, may be morbidly exalted, and yet the muscles be incapable of obeying the will, as often happens in paralysis." This statement is, then, illustrated under a great variety of aspects, according to the distribution of nerves and the causes that may institute reflex actions upon the organs of organic life. Examples of this nature are of constant occurrence, particularly from page 283 to 380. Many others are interspersed from the beginning of the article upon "Structure," at page 50 to the end of the volume. As one of these more casual examples, we may quote the following specification of the nervous medium through which our author interprets the operation of remedies upon distant parts when applied to the skin, and which covers, essentially, the whole ground claimed by Dr. Campbell and Dr. Allen. In introducing the modus operandi of "Counter-Irritants," Dr. Paine remarks that "I enter now upon the consideration of those remedical agents which establish their influences upon internal organs through the medium of the skin; and here is open to us a display of those sympathetic processes which take their origin in cerebro-spinal nerves along with the sensitive fibres of the sympathetic, and terminate in the motor fibres of the ganglionic system."—Page 642.

We shall not now pursue any further the subject of the "excito-secretory function." If what we have shown at present and in former articles be not satisfactory to our Nashville critic, he will doubtless remain alone in his incredulity. To those who are in possession of Dr. Paine's writings we may owe the apology of saying, that our articles have not been in the least intended to expound or to indicate what must be at once obvious to every reader, but we have hoped that we might be instrumental in correcting errors in other quarters, and in directing attention to the greatly meritorious labors of our countryman. Had our Nashville critic looked a little more deeply into the "Institutes," he would have seen that Dr. Paine sets up no claim to the discovery of the "excito.secretory function." On the contrary, he says that the function was discovered by Wilson Philip as early as 1815, and he quotes from Philip's experiments, as contained in the London Philosophical Transactions, for the purpose of applying Dr. Philip's demonstrations of the physiological influences of the nervous system upon the secretions to pathology and therapeutics, and which may be readily found by our critic on consulting the second index, articles *Secretion and Excretion*, and *Reflex Action*. We would also commend to our critic's attention other articles contained in that index, and the references which they embrace, particularly *Sweat*, *Lactation*, *Bile*, *Urine*, *Uterus*, *Youth*, *Nervous Power*, *Nervous System*, *Mental Emotion*, *Alteratives*, *Remedial Action*, *Blood-letting*. Dr. Paine claims, however, and very justly, as may be seen by our extracts, a long priority in designating the nervous mechanism through which the secretions are physiologically influenced; and although he has not thought it worth his while to insist upon his priority in the small matter of bestowing a name upon the function, we have shown that he suggested the very name which is now apparently conceded, by nearly all the medical periodicals in this country, to form the only originality belonging to Dr. Campbell. But what is alone of any practical importance; Dr. Paine was not only the first, but still the only one to carry the "excito-secretory function" and all the physiological laws of the nervous system into pathology and therapeutics.

But after all, the whole of this disputation has its origin in a mere pretense that has grown out of a name. "*Excito-secretory function*" is the magic word which is made to engulph the whole philosophy that concerns the labyrinth of the organic functions in their connection with the nervous system. But it is a word of such partial import as not to convey the slightest connection with pathology and therapentics, but, on the contrary, to impress the belief that is is limited to the natural state of the body. It disregards all the modifying influences of the nervous system upon organic actions and their products, whether induced by remedial or morbific agents, and the inappropriateness of the term, beyond its mere physiological import, may be readily seen, should any one attempt its introduction into any of the pathological or therapeutical branches of Dr. Paine's "Institutes of Medicine." It would produce a confusion that would render them unintelligible. Dr. Allen, in his reclamation, had the sagacity to see this, when he says, in nearly Dr. Paine's language, "*The influence is not confined to the mere increase of action*, as the term *excitor* might perhaps suggest. The reverse may take place, The excitor may rather become the *depressor*. It would be as correct to say the depressor-motory, the depressor secretory, as to say the excitor idem."

Our critic, in this state of ignorance, asks why Dr. Paine did not earlier set up his claim to originality in the matter of the foregoing name aad function. Besides the reasons just assigned, his works had been many years in the hands of the profession, and, as we imagine, he very naturally supposed that his medical brethren would spontaneously recognize his rights. This reason, indeed, is assigned by Dr. Paine himself; aud if our critic had not felt that he was employed in a hopeless effort, he would have quoted Dr. Paine's avowal of his reliance upon that common sense of justice, which sooner or later dispenses its judgments in the righteousness of truth. In his essay on the "Rights of Authors," he says, "The author has relied upon his professional brethren for ultimate justice, '*ultimum et unicum remedium, jus aliqnando dormitvr, moritur nonquam.*' But the author has lately seen so great an indisposition in certain quarters to allow him any credit for his labors, that he has concluded to make this expostulation, which refers particularly to the following dispute about the authorship ot matters in which neither of the gentlemen has any interest, but the anthor of these Institutes."—Page 913.

Our critic, in the infirmity of his cause, devotes much of his article to an obvious typographical error which occurs in our communication to the Medical Gazette, to which we are made to say that "there is scarcely an idea in his (Dr. Campbell's) essay but what may be found in the writings of Dr. Paine, *or* has been advanced in his annual lectures for the last seventeen years," the word *and* instead of *or* having been written in our manuscript; nor will our critic entertain any doubt of this statement, or that we intended to say that the whole compass of Dr. Campbell's ideas was embraced in Dr. Paine's *writings*, after he shall have read the present article, though we would commend to his attention the extracts which we have made from the same writings in our article published in the New York Journal of Medicine. Lest, however, our critic should still avail himself of typograpical errors, we will anticipate this infirmity by stating that no less than *tweuty-nine* appear in our article in that periodical. C. A. L.

*American Med. Gazette.*

